# Arterial Hemodynamics in Prehypertensives

**DOI:** 10.1155/2019/3961723

**Published:** 2019-04-01

**Authors:** Chih-Tai Ting, Jaw-Wen Chen, Mau-Song Chang, Frank Chi-Pong Yin

**Affiliations:** ^1^Cardiovascular Center, Taichung Veterans General Hospital, Taichung, Taiwan; ^2^Department of Medical Research, Veterans General Hospital, Taipei, Taiwan; ^3^Department of Medicine and Cardiovascular Research Center, National Yang Ming University School of Medicine, Taipei, Taiwan; ^4^Cardiology Division, Department of Medicine, Veterans General Hospital, Taipei, Taiwan; ^5^Department of Biomedical Engineering, Washington University in St. Louis, St. Louis, MO, USA

## Abstract

Compared to age-matched normotensive adults, those with essential hypertension have been shown to have distinct arterial hemodynamic abnormalities consisting of increased peripheral resistance, pulse wave velocity, and wave reflection magnitude as well as decreased wave reflection time and aortic compliance. These abnormalities are further exacerbated by beta-adrenergic blockade. To see if there are similar hemodynamic abnormalities that antedate the onset of fixed hypertension, we compared age-matched normotensives with prehypertensives selected from patients undergoing diagnostic cardiac catheterization. Ascending aortic pressure and flow were measured with a micromanometer and flow velocity sensor in the baseline state and after beta-adrenergic blockade. In the baseline state the prehypertensive compared to the normotensive group had elevated blood pressure, resistance, left ventricular end-diastolic pressure (LVEDP), and wave reflections. Beta-adrenergic blockade increased resistance, LVEDP, and wave reflections in both groups. Some of these findings are the same as those we previously reported in young persons with established, essential hypertension. The differences in LVEDP and wave reflections, both in the baseline state and after beta-blockade, were still present in subgroups with no differences in blood pressure. Hence, the elevated wave reflections in prehypertensives do not appear to be directly related to the level of blood pressure. These results support the notion that the elevated blood pressure in hypertension may represent a later manifestation of an already abnormal vascular system rather than the vascular abnormalities resulting from hypertension. Consequently, even before blood pressure becomes elevated, early diagnosis and treatment of the vascular abnormalities in prehypertensives may be warranted.

## 1. Introduction

Both invasive and noninvasive studies have documented distinct hemodynamic abnormalities in people with essential hypertension compared with age-matched normotensives [1–6]. Although there are some minor differences in findings, the consensus is that, compared to normal, peripheral resistance (R), characteristic impedance (Z_c_), pulse wave velocity (PWV), and wave reflection magnitude are increased and aortic compliance and wave reflection time are decreased. Acute administration of nonvasodilating adrenergic blockers and angiotensin converting enzyme inhibitors lowers blood pressure but does not normalize all the vascular properties. In contrast, administering a nonspecific smooth muscle dilator, nitroprusside, or a calcium-channel blocker lowered the blood pressure to the same extent but completely normalized the vascular abnormalities [1]. Therefore, these observations suggest that, in the early stage of essential hypertension, some hemodynamic abnormalities cannot be attributed, per se, to the elevated blood pressure but rather are a manifestation of reversible dysfunction in the muscular arteries modulated by smooth muscle.

Given the above observations, it is reasonable to ask whether the hemodynamic abnormalities are a consequence of or antedate the increased blood pressure. Answering this question is difficult once hypertension becomes manifest because acutely lowering the blood pressure may not be sufficient to reverse some of the longstanding neural or humoral vascular effects. If there is clear evidence of vascular abnormalities before the blood pressure becomes chronically elevated, this would help clarify this important “chicken vs. egg” issue. If chronically elevated blood pressure eventually causes vascular and target organ abnormalities, then treating the blood pressure in hopes of preventing, or even reversing, potential further damage makes sense. If, on the other hand, vascular changes antedate the onset of blood pressure increases, it might make sense to diagnose and perhaps treat the vascular abnormalities as early as possible so as to either prevent or ameliorate the later manifestations of increased blood pressure. In fact, treating the blood pressure at a later stage might be too late to reverse some of the early vascular abnormalities.

Comparing vascular function in completely normal persons with that in prehypertensives who have a strong family history and likelihood of later developing fixed hypertension but who are not yet hypertensive is one way to directly address the “chicken or egg” issue. This is the rationale for the present study. We obtained acending aortic high-fidelity micromanometric pressure and flow data during cardiac catheterization in a group of prehypertensives and a group of age-matched normotensives. We compared R, aortic impedance, pulse wave reflection magnitude, wave reflection travel time, and compliance during baseline conditions and after acute beta-adrenergic blockade with intravenous propranolol. The results demonstrate that, compared to normotensives, prehypertensives have mildly but statistically significantly higher blood pressure, LVEDP, wave reflections, and R—abnormalities very similar to those previously found in established essential hypertension. More importantly, the elevated wave reflections and LVEDP are present even in subgroups with matched blood pressure. Hence, the presence of early vascular abnormalities in prehypertension together with the increasing recognition of its deleterious effects and predilection for progressing to hypertension [7, 8] suggests that we reconsider our approach.

## 2. Materials and Methods

### 2.1. Patient Selection

The study population was selected from ethnic Chinese who were undergoing diagnostic cardiac catheterization for chest pain syndrome, evaluation of a systolic murmur, or electrophysiological evaluation. Exclusion criteria included the following: (1) evidence of congenital, coronary, or valvular heart disease; (2) age under 18; (3) pregnancy; (4) taking of medications that could affect blood pressure such as oral contraceptives, pain relievers, and antidepressants; (5) diabetes mellitus (based on fasting blood glucose level); (6) abnormal renal function based on renal arteriograms and abnormal levels of serum electrolytes, creatinine, blood urea nitrogen, and 24-hour creatinine clearance; (7) abnormal levels of cortisol, 17-ketosteroids, 17-hydroxycorticosterone, aldosterone, plasma renin activity, thyroid stimulating hormone, triiodothyronine, and free thyroxine. Based on multiple outpatient and in-hospital precatheterization standard syphgmomanometric blood pressure measurements, the patients were classified into the normotensive (N) or prehypertensive (P) main groups according to the 2003 Joint National Committee on Prevention, Detection, Evaluation, and Treatment of High Blood Pressure report (JNC 7) [9]. Those in the N main group had no instances of elevated blood pressure and no family history of hypertension. Those in the P main group had at least one instance of abnormally high blood pressure that normalized during hospitalization and the majority had unequivocal family histories of hypertension. None had a history of being treated with antihypertensive medications. All patients gave informed consent for the investigative portion of the study which was performed with the approval of and according to the guidelines of the hospital's human investigation committee.

### 2.2. Data Acquisition

All studies were performed as previously reported [10]. Briefly, patients were premedicated with 5 mg intramuscular chlorpheniramine maleate. After completion of the diagnostic portion of the catheterization, baseline high-fidelity left ventricular pressure, ascending aortic pressure, and flow velocity were first recorded for offline analysis. To assess the role of nonspecific beta-adrenergic blockade and to minimize beta-adrenergically mediated peripheral vasodilation, we intravenously administered propranolol at a rate of 1 mg/min until a dose of 0.15 mg/kg had been delivered. Hemodynamic measurements were repeated immediately on completion of beta-blockade.

### 2.3. Calculations and Data Analysis

Calculations and data analysis methods are identical to those previously reported [10]. Briefly, the pressure and flow signals were digitized at a rate of 250 Hz and resolved into their Fourier harmonics from which input impedance modulus and phase angle, R, Z_c_, the frequency of the first zero crossing of the impedance phase angle (f_0_), total external power, oscillatory power, and the ratio of oscillatory to total power were calculated. We calculated the amplitudes of the forward (P_f_) and backward (P_b_) waves and used the ratio P_b_/P_f_ as an index of wave reflection. Finally, since our previous studies have revealed concordant changes in compliance calculated at the different pressure values, for this study we restricted attention to the compliance at peak systolic pressure.

### 2.4. Statistical Analysis

For statistical analysis we used a two-way, mixed-factorial repeated measures analysis of variance (ANOVA) with the* between-subjects* factors being normotensive and prehypertensive and* within-subjects* factors being baseline and after propranolol. Statistical significance was considered to be* P*<0.05. We first examined all parameters in each main group for each condition for equality of variances using Levene's test and applied an appropriate correction for subsequent analysis only if the variances were not equal. Next, only those variables that exhibited significant* between-subjects* effects were subjected to further analysis, i.e., to discern if there were baseline differences, an effect of beta-blockade, and whether that effect differed between the main groups. Any parameter that had both significant* between-* and* within-subjects* effects was examined for an interaction effect. If no significant interaction was present, the simple main effects were compared. If there was a significant interaction, further analysis was performed to uncover any specific significantly different pairwise comparisons.

## 3. Results

### 3.1. Results

#### 3.1.1. Clinical Characteristics: Table 1

The left section of Table 1 shows the clinical characteristics of the N and P main groups. Nine of the 12 patients in the P group had an unequivocal family history of hypertension. Although the proportion of women in the N group was greater than in the P group, there was no statistically significant difference in age, body length, body weight, or aortic cross-sectional area. The corresponding data for the subgroups are shown in the right section of the table and similarly indicate no statistically significant differences between the subgroups.

#### 3.1.2. Cardiovascular Parameters: Table 2

The left section in Table 2 shows the pertinent cardiovascular parameters for the main groups in the baseline state and after propranolol. The right section shows the results for the subgroups. The baseline hemodynamic data for the N group have been reported previously [10] but are included here for completeness and ease of comparison. The data after propranolol have not been previously reported. Even restricting attention to only those parameters with significant* between-subjects* effects, there are so many pairwise comparison results with differing levels of statistical significance that the results of the statistical analysis are presented in a separate table for clarity.

#### 3.1.3. Statistical Analysis Results: Table 3

The relevant results of the statistical analysis are shown in Table 3. Only the results for those parameters with statistically significant* between-subjects *effects are included. In the baseline state, compared to N, the P main group had significantly higher systolic and diastolic aortic blood pressure, R, LVEDP, P_b_, and P_b_/P_f_. In both groups, as expected, beta-blockade significantly decreased heart rate and increased LVEDP and R. Beta-blockade also increased both P_b_ and P_b_/P_f_ in both groups with the differences between groups remaining significant. A small but statistically significant increase in systolic blood pressure (SBP) occurred only in the P group after beta-blockade.

Because the P main group had slightly, but statistically significantly, higher blood pressures than the N main group, some or all of the above results might be attributable to the pressure differences. To directly examine this possibility within each of the N and P main groups we selected subgroups whose blood pressure was closer. Specifically, we separately analyzed those in the N group with peak SBP ≥115 and those in the P group with SBP ≤ 130. The results of the subgroups analyses are shown in the right sections of Tables 1–3. As expected, there were no differences in baseline blood pressure between the subgroups. Despite this, LVEDP, P_b_, and P_b_/P_f_ are significantly higher and HR is significantly lower in the P than in the N subgroup, both before and after beta-blockade.

**Figure 1:**
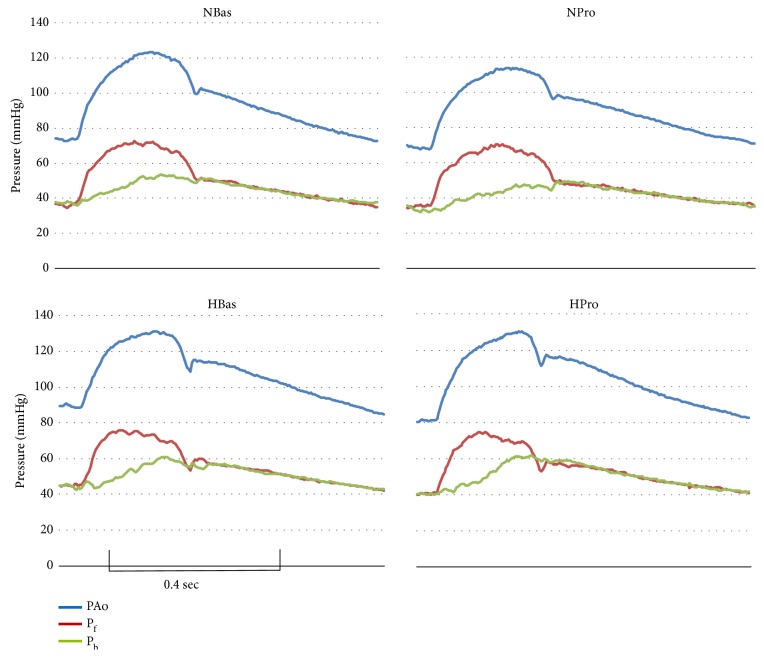
Representative plots of aortic pressure and its forward and backward components from one beat during baseline (Bas) and after propranolol (Pro) of one patient from the N subgroup and one from the P subgroup. The corresponding P_f_ (mmHg), P_b_ (mmHg), and P_b_/P_f_ values for each beat are as follows: NBas: 37.9, 17.4, and 0.46; PBas: 34.0, 18.3, and 0.54; NPro: 36.3, 17.6, and 0.48; PPro: 34.7, 21.8, and 0.63.

Figure 1 shows plots of ascending aortic pressure and its forward and backward components for one beat during baseline and after propranolol for a patient from the N subgroup and one from the P subgroup. Although they are small, there are clear differences between P_b_ in the N and P patients. For the N patient the small P_b_ that rises throughout systole to a broad dome under both conditions adds to the dome-shaped P_f_ to produce a similar dome-shaped pressure wave that peaks in late systole. In contrast, for the P patient, the decrease in P_f_ beginning early in and continuing through the rest of systole is offset by a larger P_b_ that rises to a distinct peak resulting in a composite wave with a prominent late systolic peak, especially after propranolol.

## 4. Discussion

The novelty of this study is the detailed hemodynamic characterization of a group of young prehypertensives who have nearly normal levels of blood pressure and consequently have never been treated, either acutely or chronically, with any antihypertensive medications. There are three major findings which provide some new insights into prehypertension. Compared to pressure- and age-matched normotensives, prehypertensives have (1) elevated wave reflections, (2) no difference in wave reflection travel time, and (3) elevated LVEDP. This strongly suggests that these abnormalities are not directly attributable to the level of blood pressure. To our knowledge, this is the first direct evidence of the dissociation between wave reflections and blood pressure. Several previous studies provide corroborating indirect evidence of this dissociation. In more than 40 similarly aged ethnic Chinese with blood pressure much higher than in our P main group, the range of baseline P_b_/P_f_ was in the same range as we found [1]. When either nitroprusside or a calcium-channel antagonist was administered to hypertensives, despite the blood pressure decreasing significantly—but still remaining higher than normal—the wave reflection index completely normalized. When normotensives and hypertensives performed handgrip exercise, despite the systolic blood pressure increasing by about 20 mm Hg in both groups, there was no effect on the wave reflection index [11]. Another study reported dissociation between exercise-induced blood pressure changes and wave reflections in treated hypertensives [12].

There is increasing evidence in muscular arteries of the important role of smooth muscle in modulating wave reflections. The fact that elevated wave reflections and central blood pressure augmentation can be normalized with dilating as compared with nondilating antihypertensive drugs [1, 13, 14] demonstrates that the enhanced wave reflections are not due to fixed structural entities. The increase in wave reflections after beta-blockade observed in both the N and P groups of this and a previous study [15] is consistent with previous results implicating modulation by the autonomic nervous system [3, 16]. There is also increasing evidence for a role of the endothelium in the elevation of wave reflections. Limited nitric oxide availability was reported in hypertensives [17]. Two recent studies reported an association between specific eNOS gene polymorphisms and several abnormal indices of vascular function in age-matched normotensives and prehypertensives [18, 19] thereby providing a mechanistic basis for the vascular changes. A similar level of endothelial damage was observed in young prehypertensives as compared to age-matched hypertensives [20]. As has been emphasized, however, endothelial dysfunction alone is not necessarily associated with hypertension [17]. For endothelial dysfunction to be deleterious its manifestations must negatively impact other regions of the cardiovascular system—such as via wave reflections.

In the arterial system reflections arise from aortic tapering, branch points, and adjacent regions of differing stiffness. The specific site(s) of reflection are, however, difficult to pinpoint. There appears to be a major reflection near the renal arteries as well as near the iliac bifurcation [21]. In contrast, a modeling study demonstrated nearly equal reflections from the proximal and distal aortic regions [22]. Regardless of site of origin, the reflections merge to produce the resultant central aortic reflected pressure wave whose effects depend critically on morphology, magnitude, and timing [4, 12, 14, 22–24]. If the reflection time is sufficiently long so that the bulk of the backward wave arrives in the ascending aorta during diastole the reflections would have little impact on cardiac loading. Conversely, if reflection time is sufficiently short so that the bulk of the reflected wave arrives during systole, peak and pulse pressure will be increased which will be detrimental to the heart and other organs.

In the present study, unlike in fixed hypertensives [1], we found no significant difference between the groups in f_0_. This is not altogether surprising since the major determinants of f_0_ are arterial pulse wave velocity (PWV) and/or the effective wave reflecting site [25]. Even though we did not directly measure PWV, the normal value of f_0_ and the mildly elevated blood pressure in the P main group strongly suggests that PWV was not substantially elevated in the prehypertensives. If either PWV were sufficiently increased or the reflecting site were sufficiently proximal in the presence of elevated wave reflections, we would have observed a more marked elevation of central systolic blood pressure.

The small but significant increase above normal in LVEDP in both our P main group and subgroups is evidence for an early, subtle alteration in cardiac function. Since elevated blood pressure with its attendant increased stresses on the heart does not appear to be responsible, it is reasonable to ask how abnormally high wave reflection but normal wave reflection time can be deleterious. It is plausible that some daily activities—such as those involving isometric exercise or even mental stress—transiently increase blood pressure which in turn increases PWV and thereby sufficiently shortens wave reflection time to affect systolic function. Over a prolonged period, without needing to invoke chronic mechanisms such as fixed vascular damage or anatomic abnormalities, this transient loading could be deleterious. There is some indirect evidence supporting this contention. Handgrip exercise in both normotensives and hypertensives shortened wave reflection time without affecting P_b_/P_f_ [11]. PWV during handgrip exercise, independent of wave reflection magnitude, was found to be the strongest predictor of LV mass index in treated hypertensives [12]. In young men with low, normal, and high normal blood pressure, a mental stress challenge induced significantly different increases in both blood pressure and catecholamines that were directly related to the baseline pressure levels [16]. In contrast, wave reflection magnitude, but neither PWV nor wave reflection time, was found to be the strongest predictor of LV mass regression during a year of treatment in hypertensives [24]. The lack of effect of PWV could be because the already elevated PWV blunted any additional effect. Another study in a general population of men and women found that aortic compliance, peripheral resistance, and reflected wave magnitude were independent predictors of increased LV mass [26]. That study, however, did not examine the effect of wave reflection time. In addition to wave reflections, there is other evidence of abnormal ventricular diastolic function as manifested in prolonged isovolumic relaxation time and slower filling in prehypertensives compared to normotensives [27].

These short-term, reversible effects of wave reflections will also be affected by chronic effects. One example is aging which is well known to increase stiffness of the large arteries. The resultant increase in PWV shortens reflection time and thereby increases central arterial systolic and pulse pressure [28, 29]. The former puts an additional load on the heart and the latter transmits pressure waves deeper into target organs. Moreover, the increased arterial pressure distends the large arteries causing an even greater increase in stiffness—resulting in a deleterious vicious cycle. In prehypertensives with already elevated wave reflections these deleterious effects could not only begin earlier but also be more pronounced with aging than in normotensives. Indeed, some long-term effects of elevated wave reflections have been reported [4, 30]. In the latter study, an elevated P_b_—independent of heart rate, age, height, gender, or PWV—was found to be a strong predictor of long-term cardiovascular mortality.

In addition to the parameters already discussed, we found the HRs of the P compared to the N main groups and subgroups to be slightly but significantly lower, both at baseline and after beta-blockade. This differs from other studies that found an increased HR in prehypertensives compared to age-matched normotensives [27, 31]. The substantially different study conditions as well as differing ethnicity of the study groups could be reasons for these different findings. Regardless, by affecting the durations of systole and diastole, HR could directly affect f_0_. However, because of the small differences in HR there is unlikely to be a discernible effect. In fact, we found no differences between any of the groups in f_0_. HR differences are unlikely to impact the other parameters which differed between the N and P groups. Resistance is independent of time (and hence HR) because it is a ratio of two factors, both of which are time dependent. P_b_ is a magnitude that is independent of time, and P_b_/P_f_ is also a ratio. Although we do not know the reason(s) for the lower HR in the P group, it might be related to the fact that, compared to age-matched normotensive counterparts, male but not female prehypertensives have been found to have abnormal autonomic control of heart rate and increased sympathovagal imbalance [27, 31]. The abnormal adrenergically mediated smooth muscle function could be another manifestation of autonomic dysfunction in prehypertension.

There are some limitations of our study that deserve discussion. First, we categorized patients based on the JNC 7 classification scheme in play at the time of the study [9]. According to the new 2017 guidelines [32], however, the patients in our P groups would now be reclassified into elevated or stage 1. Since blood pressure is a continuum any categorization is rather arbitrary. Hence, for the sake of simplicity and consistency we used the JNC 7 classification. Second, it is highly likely that the anxiety of the procedure caused the blood pressure reported herein of some, or many, of the patients to be higher than the precatheterization values on which they were categorized. We used this categorization to avoid the vagaries associated with categorizing based on the blood pressure at the time of the procedure but doing so may have made delineation of the categories a bit uncertain. Third, there are many noninvasive methods to estimate central aortic pressure and flow [3–6]. Each of these, however, entails an approximation, assumption, or mathematical transformation which has been validated but still engenders a certain degree of uncertainty. Instead, we used invasive measurements which are the most direct and accurate but which, admittedly, limited the study to a very small number of patients. The fact that we found statistically significant differences between groups, however, attests to the high quality, consistency, and robustness of the data. Fourth, our findings pertain to acute changes in young people in an environment far from normal. Whether similar results would be found with much larger numbers of people spanning a wider age range and in more normal settings clearly needs to be determined. Fifth, females comprised a smaller proportion than males in all groups so any specific gender effect would have been masked by the larger number of males. Additionally, there were proportionally more females in both the N compared to P main groups and subgroups. One parameter most likely to be affected by this gender imbalance is f_0_ because, in general, women tend to have shorter body lengths than men and body length is a factor potentially affecting wave reflection time. Indeed, of the five shortest body lengths in our population, four were women. In addition, unlike men, women prehypertensives did not have abnormal autonomic control of heart rate nor sympathovagal inhibition [27, 31]. Consequently, to directly examine this issue, we excluded females from all the main and subgroups and performed statistical analysis of f_0_, as well as all the parameters listed in Table 3. This additional analysis revealed identical conclusions as when females were included (data not shown). Hence, the gender imbalance of our groups does not affect our conclusions.

Finally, with respect to future directions and therapeutic implications, our findings of increased wave reflections and subtle cardiac effects in young prehypertensives could be only the tip of the iceberg. It seems clear that further large-scale studies in prehypertensives, focusing specifically on hemodynamics during provocations such as exercise, are warranted to more clearly elucidate the pathophysiology of this condition.

Independent of our findings, there is also increasing evidence for deleterious effects of prehypertension. For example, one study found a familial disposition for hypertension across three generations, especially with early onset (< age 55) hypertension in grandparents [33]. Among young, normotensives parental hypertension was associated with increased arterial stiffness, wave reflections, and aortic augmentation index [34]. A four-year cumulative incidence of progression of nonhypertensives to hypertensives was found to increase stepwise across optimal, normal, and high normal blood pressure groups [35]. Prehypertension is statistically significantly associated with target organ damage, not only in the heart but also, especially, in the brain and kidneys [7]. Meta-analysis of a cohort study reported an elevated risk ratio of coronary heart disease in high normal pressure prehypertensives but not in the low normal pressure group [36]. Both gender and age-related increases in cardiovascular disease incidence in high normal pressure prehypertensives have been reported [37]. There appears to be a modest negative association between blood pressure and cognitive function [38]. Finally, a study of more than 2 million Israeli adolescents followed for an average of 17 years revealed that those in the normal-to-high-normal prehypertensive range had increased incidence of adult end-stage renal disease with a hazard ratio of 1.32 [39].

Current guidelines do not suggest treating prehypertensives. However, in light of the findings discussed above and if our results are borne out by further studies, there will be more compelling evidence of a need to reconsider our approach to prehypertension. This is especially germane since it appears that the hemodynamic abnormalities in young persons with prehypertension or established essential hypertension are still reversible. In particular, it might be worth considering alterations in lifestyle and the early use of specific classes of antihypertensive drugs that act to reduce wave reflections.

## 5. Conclusion

During diagnostic cardiac catheterization we measured detailed aortic hemodynamics in normotensives and age-matched prehypertensives in the baseline state and after acute beta-adrenergic blockade. In the baseline state the prehypertensives compared to the normotensives had elevated blood pressure, resistance, LVEDP, and wave reflections. Beta-adrenergic blockade increased resistance, LVEDP, and wave reflections in both groups. In subgroups selected so that there were no differences in blood pressure, the differences in LVEDP and wave reflections in the baseline state and after beta-blockade were still present. These baseline vascular abnormalities and responses to beta-blockade are very similar to those we previously reported in young persons with established, essential hypertension. Importantly, the elevated wave reflection in prehypertensives with the same blood pressure as normotensives suggests that this abnormality is not directly related to the level of blood pressure. Hence, these results support the notion that the elevation of blood pressure in hypertension may represent a later manifestation of an already abnormal vascular system rather than the vascular abnormalities being a result of the hypertension. Some implications for morbidity and treatment are discussed.

## Figures and Tables

**Table 1:** Clinical characteristics (mean ± SD) of the entire study population comprised of the N and P main groups and subgroups.

	Main groups		Subgroups	
Parameter	Normotensives (N)	Prehypertensives (P)	Normotensives (N)	Prehypertensives (P)
Number	14	12	7	7
Male/female	10/4	11/1	5/2	7/0
Positive family history	0	9	0	4
Age (yrs)	32.4 ± 7.5	33.4 ± 5.7	34.0±6.5	33.0±7.3
Body length (cm)	167.3± 9.0	167.8 ± 7.6	166.4±9.9	171.4±4.6
Body weight (kg)	66.9 ± 12.5	72.3 ± 5.7	68.3±13.3	73.4±6.9
Aortic CSA (cm^2^)	6.02 ± 1.36	7.13 ± 1.89	6.73±1.12	6.83±2.26

P=NS for all N vs. P parameters.

**Table 2:** Baseline (Bas) and after-propranolol (Pro) hemodynamic parameters (mean ± SD) in the main groups and the subgroups.

	Main groups				Subgroups			
Parameter	Normotensives (N) (n=14)		Prehypertensives (P) (n=12)		Normotensives (N) (n=7)		Prehypertensives (P) (n=7)	
	Bas	Pro	Bas	Pro	Bas	Pro	Bas	Pro
HR	83.5±13.5	74.3±9.7	76.1±6.1	67.3±4.0	89.6±13.7	76.6±10.4	74.2±5.2	66.6±4.1
SV	74.9±15.0	75.8±17.6	79.9±11.9	75.6±16.3	73.3±6.9	75.8±8.5	80.1±9.7	73.6±12.5
LVEDP	10.2±4.1	13.8±3.4	17.7±4.2	20.3±4.8	10.6±4.4	13.9±3.6	18.7±4.7	19.6±5.8
SBP	111.8±11.3	113.5±10.6	129.1±6.7	133.2±10.0	120.6±5.5	119.9±7.3	125.4±5.7	128.6±9.5
DBP	73.5±9.9	74.0±9.7	84.5±5.8	86.1±7.3	80.0±4.5	78.8±7.1	81.4±5.0	83.0±7.5
R	1239±224	1402±296	1435±266	1788±434	1257±219	1410±267	1414±177	1763±338
Z_c_	75.5±21.5	69.0±20.5	70.8±17.3	72.0±18.2	72.7±14.4	66.1±13.9	75.2±16.1	78.9±16.3
W_t_	1497±347	1383±441	1687±346	1412±340	1693±234	1471±195	1596±264	1314±241
W_o_/W_t_	0.15±0.03	0.14±0.03	0.14±0.02	0.13±0.02	0.15±0.02	0.14±0.03	0.14±0.02	0.13±0.03
P_f_	31.9±3.8	30.5±3.7	33.1±4.9	32.1±4.2	33.4±3.8	31.4±4.6	33.4±4.4	32.5±4.8
P_b_	13.8±2.5	15.6±2.5	18.5±2.4	20.8±3.4	14.4±2.8	15.8±3.0	18.2±2.3	20.1±3.7
P_b_/P_f_	0.43±0.07	0.51±0.06	0.56±0.06	0.65±0.08	0.43±0.08	0.51±0.08	0.55±0.06	0.63±0.10
f_0_	3.1±0.6	3.3±0.6	3.6±0.7	3.8±0.8	3.5±0.5	3.5±0.3	3.5±0.6	3.6±0.9
C_s_	1.8±0.8	1.7±0.8	1.4±0.4	1.2±0.4	1.6±0.5	1.5±0.4	1.4±0.4	1.3±0.4

Abbreviations: HR = heart rate (bpm); SV = stroke volume (ml); LVEDP = left ventricular end-diastolic pressure (mmHg); SBP/DBP = peak systolic/diastolic aortic blood pressure (mmHg); R = peripheral resistance (dyne-sec/cm^5^); W_t_ = total external power (milliwatts); W_o_ = oscillatory external power (milliwatts); Z_c_ = characteristic impedance (dyne-sec/cm^5^); P_f_ = magnitude of forward aortic pressure component (mmHg); P_b_ = magnitude of backward aortic pressure component (mmHg); f_0_ = first zero-crossing of aortic impedance modulus (Hz); C_s_ = aortic compliance at peak systolic blood pressure (ml/mmHg).

**Table 3:** Statistical results of only those parameters in Table 2 with statistically significant between-subjects effects for the main groups and subgroups.^*∗*^

	Main groups				Subgroups			
Parameter	NBas-PBas	NBas-NPro	PBas-PPro	NPro-PPro	NBas-PBas	NBas-NPro	PBas-PPro	NPro-PPro
HR		.001	<.001	.03	.017	<.001	.001	.04
LVEDP	<.001	.01	.03	.001	.01	.04		.05
SBP	<.001		.01	<.001				
DBP	.002			.002				
R	.05	.01	<.001	.01				
P_b_	<.001	.002	.001	<.001	.02	.002	.04	.03
P_b_/P_f_	<.001	<.001	.003	<.001	.01	.001	.03	.02

^*∗*^The *P* values listed are for simple main effects except that R for the entire population and HR for the subgroups had significant *within-* and* between-subjects* interaction effects. The *P* values from pairwise comparisons for those two sets of data are shown.

## Data Availability

All of the available data are included in the article.

## References

[1] Ting C.-T., Chen C.-H., Chang M.-S., Yin F. C. P. (1995). Short- and long-term effects of antihypertensive drugs on arterial reflections, compliance, and impedance. *Hypertension*.

[2] Li W., Ahn A. C. (2012). Pulsatile hemodynamics of hypertension: systematic review of aortic input impedance. *Journal of Hypertension*.

[3] Saba P. S., Cameli M., Casalnuovo G. (2014). Ventricular-vascular coupling in hypertension: methodological considerations and clinical implications. *Journal of Cardiovascular Medicine*.

[4] Adji A., O’Rourke M. F., Namasivayam M. (2011). Arterial stiffness, its assessment, prognostic value, and implications for treatment. *American Journal of Hypertension*.

[5] Laurent S., Cockcroft J., Van Bortel L. (2006). Expert consensus document on arterial stiffness: methodological issues and clinical applications. *European Heart Journal*.

[6] Mitchell G. F., Lacourciere Y., Quellet J. P. (2003). Determinants of elevated pulse pressure in middle-aged and older subjects with uncomplicated systolic hypertension: the role of proximal aortic diameter and the aortic pressure-flow relationship. *Circulation*.

[7] Materson B. J., Garcia-Estrada M., Degraff S. B., Preston R. A. (2017). Prehypertension is real and can be associated with target organ damage. *Journal of the American Society of Hypertension*.

[8] Egan B. M., Stevens-Fabry S. (2015). Prehypertension—prevalence, health risks and management strategies. *Nature Reviews Cardiology*.

[9] Chobanian A. V., Bakris G. L., Black H. R. (2003). Seventh report of the joint national committee on prevention, detection, evaluation, and treatment of high blood pressure. *The Journal of the American Medical Association*.

[10] Ting C.-T., Chen J.-W., Chang M.-S., Yin F. C. P. (1995). Arterial hemodynamics in human hypertension: effects of the calcium channel antagonist nifedipine. *Hypertension*.

[11] Ting C. T., Brin K. P., Lin S. J. (1986). Arterial hemodynamics in human hypertension. *The Journal of Clinical Investigation*.

[12] Chirinos J. A., Segers P., Raina A. (2010). Arterial pulsatile hemodynamic load induced by isometric exercise strongly predicts left ventricular mass in hypertension. *American Journal of Physiology-Heart and Circulatory Physiology*.

[13] Miyashita H., Aizawa A., Hashimoto J. (2010). Cross-sectional characterization of all classes of antihypertensives in terms of central blood pressure in Japanese hypertensive patients. *American Journal of Hypertension*.

[14] Safar M. E., Levy B. I. (2015). Studies on arterial stiffness and wave reflections in hypertension. *American Journal of Hypertension*.

[15] Ting C. T., Chou C. Y., Chang M. S., Wang S. P., Chiang B. N., Yin F. C. P. (1991). Arterial hemodynamics in human hypertension: effects of adrenergic blockade. *Circulation*.

[16] Flaa A., Mundal H. H., Eide I., Kjeldsen S., Rostrup M. (2006). Sympathetic activity and cardiovascular risk factors in young men in the low, normal and high blood pressure ranges. *Hypertension*.

[17] Taddei S., Salvetti A. (2002). Endothelial dysfunction in essential hypertension: clinical implications. *Journal of Hypertension*.

[18] Seidlerová J., Filipovský J., Mayer O., Kučerová A., Pešta M. (2015). Association between endothelial NO synthase polymorphisms and arterial properties in the general population. *Nitric Oxide: Biology and Chemistry*.

[19] Pal G. K., Adithan C., Umamaheswaran G. (2016). Endothelial nitric oxide synthase gene polymorphisms are associated with cardiovascular risks in prehypertensives. *Journal of the American Society of Hypertension*.

[20] Lyamina N. P., Lyamina S. V., Senchikhin V. N., Dodina K. A., Manukhina E. B., Downey H. F. (2011). Endothelial damage reflected by circulating endothelial cells in young patients with latent or manifest hypertension. *The FASEB Journal*.

[21] Latham R. D., Westerhof N., Sipkema P., Rubal B. J., Reuderink P., Murgo J. P. (1985). Regional wave travel and reflections along the human aorta: a study with six simultaneous micromanometric pressures. *Circulation*.

[22] Westerhof B. E., Westerhof N. (2012). Magnitude and return time of the reflected wave: the effects of large artery stiffness and aortic geometry. *Journal of Hypertension*.

[23] Torjesen A. A., Wang N., Larson M. G. (2014). Forward and backward wave morphology and central pressure augmentation in men and women in the framingham heart study. *Hypertension*.

[24] Hashimoto J., Westerhof B. E., Westerhof N., Imai Y., O’Rourke M. F. (2008). Different role of wave reflection magnitude and timing on left ventricular mass reduction during antihypertensive treatment. *Journal of Hypertension*.

[25] Latson T. W., Hunter W. C., Katoh N., Sagawa K. (1988). Effect of nitroglycerin on aortic impedance, diameter, and pulse-wave velocity. *Circulation Research*.

[26] Zamani P., Bluemke D. A., Jacobs D. R. (2015). Resistive and pulsatile arterial load as predictors of left ventricular mass and geometry: the multi-ethnic study of atherosclerosis. *Hypertension*.

[27] Pitzalis M. V., Iacoviello M., Massari F. (2001). Influence of gender and family history of hypertension on autonomic control of heart rate, diastolic function and brain natriuretic peptide. *Journal of Hypertension*.

[28] McEniery C. M., Hall I. R., Qasem A., Wilkinson I. B., Cockcroft J. R. (2005). Normal vascular aging: differential effects on wave reflection and aortic pulse wave velocity: the Anglo-Cardiff Collaborative Trial (ACCT). *Journal of the American College of Cardiology*.

[29] Hickson S. S., Butlin M., Graves M. (2010). The relationship of age with regional aortic stiffness and diameter. *JACC: Cardiovascular Imaging*.

[30] Wang K., Cheng H., Sung S. (2010). Wave reflections and arterial stiffness in the prediction of 15-year all-cause and cardiovascular mortalities: a community-based study. *Hypertension*.

[31] Pal G. K., Pal P., Nanda N., Lalitha V., Dutta T. K., Adithan C. (2013). Sympathovagal imbalance in young prehypertensives: importance of male-female difference. *The American Journal of the Medical Sciences*.

[32] Whelton P. K., Carey R. M., Aronow W. S. (2018). 2017 ACC/AHA/AAPA/ABC/ACPM/AGS/APhA/ASH/ASPC/ NMA/PCNA guideline for the prevention, detection, evaluation, and management of high blood pressure in adults: a report of the american college of cardiology/american heart association task force on clinical practice guidelines. *Hypertension*.

[33] Niiranen T. J., McCabe E. L., Larson M. G. (2017). Risk for hypertension crosses generations in the community: a multi-generational cohort study. *European Heart Journal*.

[34] Andersson C., Quiroz R., Enserro D. (2016). Association of parental hypertension with arterial stiffness in nonhypertensive offspring: the framingham heart study. *Hypertension*.

[35] Vasan R. S., Larson M. G., Leip E. P., Kannel W. B., Levy D. (2001). Assessment of frequency of progression to hypertension in non-hypertensive participants in the framingham heart study: a cohort study. *The Lancet*.

[36] Shen L., Ma H., Xiang M.-X., Wang J.-A. (2013). Meta-analysis of cohort studies of baseline prehypertension and risk of coronary heart disease. *American Journal of Cardiology*.

[37] Vasan R. S., Larson M. G., Leip E. P. (2001). Impact of high-normal blood pressure on the risk of cardiovascular disease. *The New England Journal of Medicine*.

[38] Jennings J. R., Muldoon M. F., Ryan C. (2017). Prehypertensive blood pressure and regional cerebral blood flow independently relate to cognitive function in midlife. *Journal of the American Heart Association*.

[39] Leiba A., Twig G., Vivante A. (2017). Prehypertension among 2.19 million adolescents and future risk for end-stage renal disease. *Journal of Hypertension*.

